# *N*′-(3-Methoxy­benzyl­idene)aceto­hydrazide

**DOI:** 10.1107/S1600536809028864

**Published:** 2009-07-25

**Authors:** Lu-Ping Lv, Tie-Ming Yu, Wen-Bo Yu, Wei-Wei Li, Xian-Chao Hu

**Affiliations:** a Department of Chemical Engineering, Hangzhou Vocational and Technical College, Hangzhou 310018, People’s Republic of China; b Research Center of Analysis and Measurement, Zhejiang University of Technology, Hangzhou 310014, People’s Republic of China

## Abstract

In the title mol­ecule, C_10_H_12_N_2_O_2_, the acetohydrazide group is planar within 0.012 (1) Å and forms a dihedral angle of 5.25 (8)° with the benzene ring. The meth­oxy group is coplanar with the attached benzene ring [C—O—C—C = 0.1 (2)°]. The mol­ecule adopts a *trans* configuration with respect to the C=N double bond. In the crystal, mol­ecules are linked into centrosymmetric dimers by N—H⋯O hydrogen bonds and these dimers are linked into a ribbon-like structure along [110] by C—H⋯O hydrogen bonds. In addition, an inter­molecular C—H⋯π inter­action is observed.

## Related literature

For general background to the analytical applications of Schiff bases, see: Cimerman *et al.* (1997 ▶). For their mild bacteriostatic activity and potential use as oral iron-chelating drugs for genetic disorders such as thalassemia, see: Offe *et al.* (1952 ▶); Richardson *et al.* (1988 ▶). For related structures, see: Li & Jian (2008 ▶); Tamboura *et al.* (2009 ▶).
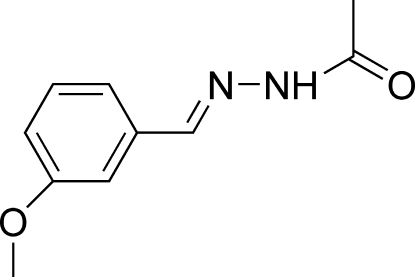


## Experimental

### 

#### Crystal data

C_10_H_12_N_2_O_2_
*M* *_r_* = 192.22
Monoclinic, 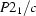

*a* = 12.394 (4) Å
*b* = 5.7278 (19) Å
*c* = 15.017 (5) Å
β = 107.126 (4)°
*V* = 1018.8 (6) Å^3^
*Z* = 4
Mo *K*α radiation
μ = 0.09 mm^−1^
*T* = 223 K
0.23 × 0.22 × 0.18 mm

#### Data collection

Bruker SMART CCD area-detector diffractometer
Absorption correction: multi-scan (*SADABS*; Bruker, 2002 ▶) *T*_min_ = 0.982, *T*_max_ = 0.985
4871 measured reflections
1768 independent reflections
1502 reflections with *I* > 2σ(*I*)
*R*_int_ = 0.025

#### Refinement

*R*[*F* ^2^ > 2σ(*F* ^2^)] = 0.040
*wR*(*F* ^2^) = 0.116
*S* = 1.07
1768 reflections
130 parameters
H-atom parameters constrained
Δρ_max_ = 0.14 e Å^−3^
Δρ_min_ = −0.15 e Å^−3^

### 

Data collection: *SMART* (Bruker, 2002 ▶); cell refinement: *SAINT* (Bruker, 2002 ▶); data reduction: *SAINT*; program(s) used to solve structure: *SHELXS97* (Sheldrick, 2008 ▶); program(s) used to refine structure: *SHELXL97* (Sheldrick, 2008 ▶); molecular graphics: *SHELXTL* (Sheldrick, 2008 ▶); software used to prepare material for publication: *SHELXTL*.

## Supplementary Material

Crystal structure: contains datablocks I, global. DOI: 10.1107/S1600536809028864/ci2861sup1.cif

Structure factors: contains datablocks I. DOI: 10.1107/S1600536809028864/ci2861Isup2.hkl

Additional supplementary materials:  crystallographic information; 3D view; checkCIF report

## Figures and Tables

**Table 1:** Hydrogen-bond geometry (Å, °)

*D*—H⋯*A*	*D*—H	H⋯*A*	*D*⋯*A*	*D*—H⋯*A*
N2—H2⋯O2^i^	0.86	2.04	2.8846 (19)	169
C1—H1*B*⋯O2^ii^	0.96	2.49	3.350 (2)	148
C3—H3⋯*Cg*1^iii^	0.93	2.83	3.544 (2)	134

Symmetry codes: (i) 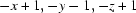; (ii) 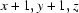; (iii) 
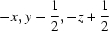
. *Cg*1 is the centroid of the C2-C7 ring.

**Fig. 1.**
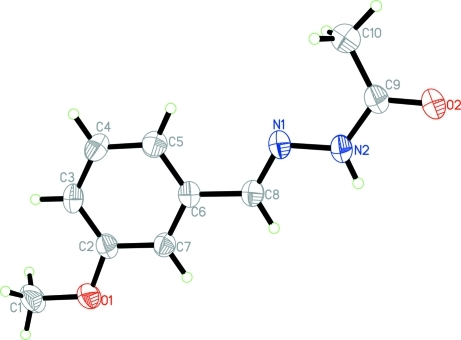
The molecular structure of the title compound. Displacement ellipsoids are drawn at the 40% probability level.

**Fig. 2.**
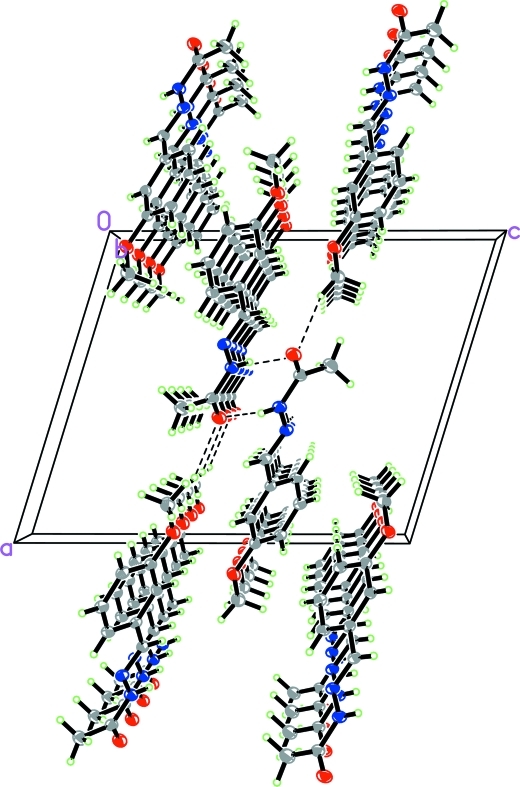
Part of the crystal packing of the title compound. Hydrogen bonds are shown as dashed lines.

## supplementary crystallographic information

### Comment

Schiff bases have attracted much attention due to the possibility of their analytical applications (Cimerman *et al.*,  1997). They are also important ligands, which have been reported to have mild bacteriostatic activity and are used as potential oral iron-chelating drugs for genetic disorders such as thalassemia (Offe *et al.*, 1952; Richardson *et al.*,  1988). Metal complexes based on Schiff bases have received considerable attention because they can be utilized as model compounds of active centres in various complexes (Tamboura *et al.*, 2009). We report here the crystal structure of the title compound (Fig. 1).

The acetohydrazide group is planar and it forms a dihedral angle of 5.25 (8)° with the benzene ring. The methoxy group is coplanar with the attached benzene ring [C1—O1—C2—C3 = 0.1 (2)°]. The molecule adopts a *trans* configuration with respect to the C═N bond. Bond lengths and angles are comparable to those observed for *N*'-[1-(4-methoxyphenyl)ethylidene]acetohydrazide (Li & Jian, 2008).

The molecules are linked by N—H···O hydrogen bonds into a centrosymmetric dimer. These dimers are linked into a ribbon-like structure along the [110] by C—H···O hydrogen bonds (Table 1 and Fig.2). In addition, an intermolecular C—H···π interaction is observed

### Experimental

3-Methoxybenzaldehyde (1.36 g, 0.01 mol) and acetohydrazide (0.74 g, 0.01 mol) were dissolved in stirred methanol (20 ml) and left for 2.5 h at room temperature. The resulting solid was filtered off and recrystallized from ethanol to give the title compound in 83% yield. Single crystals suitable for X-ray analysis were obtained by slow evaporation of an ethanol solution at room temperature (m.p. 485–487 K).

### Refinement

H atoms were positioned geometrically (N-H =  0.86 Å and C-H = 0.93 or 0.96 Å) and refined using a riding model, with *U*_iso_(H) = 1.2*U*_eq_(C,N) and 1.5*U*_eq_(C_methyl_). A rotating group model was used for the methyl groups.

### Crystal data

**Table:**

C_10_H_12_N_2_O_2_	*F*(000) = 408
*M**_r_* = 192.22	*D*_x_ = 1.253 Mg m^−^^3^
Monoclinic, *P*2_1_/*c*	Mo *K*α radiation, λ = 0.71073 Å
Hall symbol: -P 2ybc	Cell parameters from 1768 reflections
*a* = 12.394 (4) Å	θ = 1.7–25.0°
*b* = 5.7278 (19) Å	µ = 0.09 mm^−^^1^
*c* = 15.017 (5) Å	*T* = 223 K
β = 107.126 (4)°	Block, colourless
*V* = 1018.8 (6) Å^3^	0.23 × 0.22 × 0.18 mm
*Z* = 4	

### Data collection

**Table:**

Bruker SMART CCD area-detector diffractometer	1768 independent reflections
Radiation source: fine-focus sealed tube	1502 reflections with *I* > 2σ(*I*)
graphite	*R*_int_ = 0.025
φ and ω scans	θ_max_ = 25.0°, θ_min_ = 1.7°
Absorption correction: multi-scan (*SADABS*; Bruker, 2002)	*h* = −13→14
*T*_min_ = 0.982, *T*_max_ = 0.985	*k* = −6→6
4871 measured reflections	*l* = −17→17

### Refinement

**Table:**

Refinement on *F*^2^	Secondary atom site location: difference Fourier map
Least-squares matrix: full	Hydrogen site location: inferred from neighbouring sites
*R*[*F*^2^ > 2σ(*F*^2^)] = 0.040	H-atom parameters constrained
*wR*(*F*^2^) = 0.116	*w* = 1/[σ^2^(*F*_o_^2^) + (0.0591*P*)^2^ + 0.1504*P*] where *P* = (*F*_o_^2^ + 2*F*_c_^2^)/3
*S* = 1.07	(Δ/σ)_max_ = 0.001
1768 reflections	Δρ_max_ = 0.14 e Å^−^^3^
130 parameters	Δρ_min_ = −0.15 e Å^−^^3^
0 restraints	Extinction correction: *SHELXL97* (Sheldrick, 2008), Fc^*^=kFc[1+0.001xFc^2^λ^3^/sin(2θ)]^-1/4^
Primary atom site location: structure-invariant direct methods	Extinction coefficient: 0.013 (3)

### Special details

**Table:**

Geometry. All esds (except the esd in the dihedral angle between two l.s. planes) are estimated using the full covariance matrix. The cell esds are taken into account individually in the estimation of esds in distances, angles and torsion angles; correlations between esds in cell parameters are only used when they are defined by crystal symmetry. An approximate (isotropic) treatment of cell esds is used for estimating esds involving l.s. planes.
Refinement. Refinement of F^2^ against ALL reflections. The weighted R-factor wR and goodness of fit S are based on F^2^, conventional R-factors R are based on F, with F set to zero for negative F^2^. The threshold expression of F^2^ > 2sigma(F^2^) is used only for calculating R-factors(gt) etc. and is not relevant to the choice of reflections for refinement. R-factors based on F^2^ are statistically about twice as large as those based on F, and R- factors based on ALL data will be even larger.

### Fractional atomic coordinates and isotropic or equivalent isotropic displacement parameters (Å^2^)

**Table:**

	*x*	*y*	*z*	*U*_iso_*/*U*_eq_	
C1	1.15806 (15)	0.4646 (4)	0.61337 (12)	0.0650 (5)	
H1A	1.1261	0.6149	0.5928	0.097*	
H1B	1.2183	0.4331	0.5869	0.097*	
H1C	1.1870	0.4637	0.6801	0.097*	
C2	0.97784 (11)	0.3121 (3)	0.61264 (9)	0.0406 (4)	
C3	0.96000 (13)	0.4924 (3)	0.66739 (10)	0.0450 (4)	
H3	1.0137	0.6094	0.6876	0.054*	
C4	0.86019 (12)	0.4960 (3)	0.69187 (11)	0.0479 (4)	
H4	0.8475	0.6175	0.7286	0.057*	
C5	0.77987 (12)	0.3247 (3)	0.66313 (10)	0.0449 (4)	
H5	0.7136	0.3308	0.6801	0.054*	
C6	0.79857 (11)	0.1412 (2)	0.60826 (9)	0.0376 (4)	
C7	0.89771 (11)	0.1359 (3)	0.58332 (9)	0.0403 (4)	
H7	0.9108	0.0142	0.5468	0.048*	
C8	0.71693 (11)	−0.0482 (3)	0.57784 (10)	0.0407 (4)	
H8	0.7291	−0.1619	0.5376	0.049*	
C9	0.46526 (12)	−0.2813 (3)	0.59895 (10)	0.0466 (4)	
C10	0.43657 (15)	−0.1051 (3)	0.66193 (13)	0.0635 (5)	
H10A	0.3628	−0.1375	0.6674	0.095*	
H10B	0.4375	0.0484	0.6365	0.095*	
H10C	0.4911	−0.1132	0.7224	0.095*	
N1	0.62907 (10)	−0.0599 (2)	0.60576 (8)	0.0427 (3)	
N2	0.55938 (10)	−0.2483 (2)	0.57366 (8)	0.0465 (4)	
H2	0.5765	−0.3466	0.5367	0.056*	
O1	1.07271 (9)	0.2889 (2)	0.58390 (7)	0.0574 (4)	
O2	0.40548 (9)	−0.4534 (2)	0.57006 (8)	0.0591 (4)	

### Atomic displacement parameters (Å^2^)

**Table:**

	*U*^11^	*U*^22^	*U*^33^	*U*^12^	*U*^13^	*U*^23^
C1	0.0562 (10)	0.0801 (13)	0.0614 (10)	−0.0309 (9)	0.0216 (8)	−0.0073 (9)
C2	0.0391 (8)	0.0426 (9)	0.0388 (8)	−0.0071 (6)	0.0093 (6)	0.0021 (6)
C3	0.0440 (8)	0.0366 (8)	0.0478 (8)	−0.0091 (7)	0.0031 (6)	−0.0001 (6)
C4	0.0470 (9)	0.0380 (9)	0.0542 (9)	0.0020 (7)	0.0081 (7)	−0.0084 (7)
C5	0.0377 (8)	0.0437 (9)	0.0514 (9)	0.0026 (7)	0.0100 (6)	−0.0012 (7)
C6	0.0360 (7)	0.0351 (8)	0.0378 (7)	−0.0017 (6)	0.0048 (5)	0.0032 (6)
C7	0.0435 (8)	0.0387 (8)	0.0379 (7)	−0.0055 (6)	0.0108 (6)	−0.0030 (6)
C8	0.0386 (8)	0.0383 (8)	0.0422 (8)	−0.0028 (6)	0.0075 (6)	−0.0007 (6)
C9	0.0398 (8)	0.0500 (9)	0.0491 (9)	−0.0076 (7)	0.0117 (6)	0.0014 (7)
C10	0.0608 (10)	0.0665 (12)	0.0693 (11)	−0.0092 (9)	0.0285 (8)	−0.0101 (9)
N1	0.0372 (7)	0.0398 (7)	0.0481 (7)	−0.0070 (5)	0.0076 (5)	0.0003 (5)
N2	0.0399 (7)	0.0439 (8)	0.0555 (8)	−0.0116 (6)	0.0139 (5)	−0.0073 (6)
O1	0.0510 (7)	0.0670 (8)	0.0596 (7)	−0.0240 (6)	0.0249 (5)	−0.0157 (6)
O2	0.0502 (7)	0.0620 (8)	0.0693 (7)	−0.0217 (6)	0.0241 (5)	−0.0108 (6)

### Geometric parameters (Å, °)

**Table:**

C1—O1	1.4326 (19)	C6—C7	1.3863 (19)
C1—H1A	0.96	C6—C8	1.4623 (19)
C1—H1B	0.96	C7—H7	0.93
C1—H1C	0.96	C8—N1	1.2786 (19)
C2—O1	1.3730 (17)	C8—H8	0.93
C2—C3	1.378 (2)	C9—O2	1.2328 (18)
C2—C7	1.3929 (19)	C9—N2	1.3423 (19)
C3—C4	1.391 (2)	C9—C10	1.496 (2)
C3—H3	0.93	C10—H10A	0.96
C4—C5	1.374 (2)	C10—H10B	0.96
C4—H4	0.93	C10—H10C	0.96
C5—C6	1.396 (2)	N1—N2	1.3774 (17)
C5—H5	0.93	N2—H2	0.86
			
O1—C1—H1A	109.5	C6—C7—C2	120.32 (13)
O1—C1—H1B	109.5	C6—C7—H7	119.8
H1A—C1—H1B	109.5	C2—C7—H7	119.8
O1—C1—H1C	109.5	N1—C8—C6	120.94 (13)
H1A—C1—H1C	109.5	N1—C8—H8	119.5
H1B—C1—H1C	109.5	C6—C8—H8	119.5
O1—C2—C3	124.24 (13)	O2—C9—N2	119.69 (14)
O1—C2—C7	115.31 (13)	O2—C9—C10	122.14 (14)
C3—C2—C7	120.45 (13)	N2—C9—C10	118.17 (14)
C2—C3—C4	118.71 (13)	C9—C10—H10A	109.5
C2—C3—H3	120.6	C9—C10—H10B	109.5
C4—C3—H3	120.6	H10A—C10—H10B	109.5
C5—C4—C3	121.68 (14)	C9—C10—H10C	109.5
C5—C4—H4	119.2	H10A—C10—H10C	109.5
C3—C4—H4	119.2	H10B—C10—H10C	109.5
C4—C5—C6	119.47 (14)	C8—N1—N2	115.66 (12)
C4—C5—H5	120.3	C9—N2—N1	121.26 (13)
C6—C5—H5	120.3	C9—N2—H2	119.4
C7—C6—C5	119.36 (13)	N1—N2—H2	119.4
C7—C6—C8	119.09 (13)	C2—O1—C1	117.24 (13)
C5—C6—C8	121.54 (13)		
			
O1—C2—C3—C4	−179.97 (13)	C3—C2—C7—C6	0.6 (2)
C7—C2—C3—C4	−0.6 (2)	C7—C6—C8—N1	174.14 (12)
C2—C3—C4—C5	0.2 (2)	C5—C6—C8—N1	−4.7 (2)
C3—C4—C5—C6	0.3 (2)	C6—C8—N1—N2	−178.96 (11)
C4—C5—C6—C7	−0.3 (2)	O2—C9—N2—N1	−178.98 (13)
C4—C5—C6—C8	178.48 (13)	C10—C9—N2—N1	1.1 (2)
C5—C6—C7—C2	−0.1 (2)	C8—N1—N2—C9	179.20 (12)
C8—C6—C7—C2	−178.92 (12)	C3—C2—O1—C1	0.1 (2)
O1—C2—C7—C6	179.96 (12)	C7—C2—O1—C1	−179.27 (13)

### Hydrogen-bond geometry (Å, °)

**Table:**

*D*—H···*A*	*D*—H	H···*A*	*D*···*A*	*D*—H···*A*
N2—H2···O2^i^	0.86	2.04	2.8846 (19)	169
C1—H1B···O2^ii^	0.96	2.49	3.350 (2)	148
C3—H3···Cg1^iii^	0.93	2.83	3.544 (2)	134

Symmetry codes: (i) −*x*+1, −*y*−1, −*z*+1; (ii) *x*+1, *y*+1, *z*; (iii) −*x*, *y*−1/2, −*z*+1/2.
